# *Aspergillus* Infection of a Postoperative Thoracic Cavity

**DOI:** 10.1002/rcr2.70614

**Published:** 2026-05-06

**Authors:** Mio Sugino, Yusaku Kusaba, Masaaki Nagano, Shinyu Izumi

**Affiliations:** ^1^ National Center for Global Health and Medicine, Respiratory Medicine Tokyo Japan; ^2^ National Center for Global Health and Medicine, Thoracic Surgery Tokyo Japan

**Keywords:** *Aspergillus* pleurisy, bronchopleural fistula, fenestration, postoperative complication, thoracic cavity

## Abstract

A 72‐year‐old man developed *Aspergillus* infection within a postoperative thoracic cavity after lobectomy. Imaging showed cavity wall thickening with bronchopleural communication. Fenestration revealed characteristic purulent pleural debris and fungal masses. This case highlights *Aspergillus* pleurisy as a postoperative complication.

## Graphical abstract

This case describes *Aspergillus* pleurisy developing within a postoperative thoracic cavity after lung resection. Imaging revealed cavity wall thickening with bronchopleural communication, and fenestration enabled direct visualization of characteristic purulent pleural debris and fungal masses.

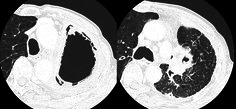

A 72‐year‐old man underwent open left upper lobectomy for Stage IIB lung squamous cell carcinoma. Postoperatively, a residual thoracic cavity remained due to incomplete lung re‐expansion. Eight months later, he developed dyspnea and fever. Chest computed tomography demonstrated irregular thickening of the inner wall of an upper left thoracic cavity lesion, and communication between the left B8 and the cavity was identified (Figure 1). Bronchoscopic sputum culture yielded *Aspergillus fumigatus*. Intravenous voriconazole was initiated; however, his condition deteriorated. On hospital day 8, fenestration was performed. Within the cavity, purulent pleural debris resembling white mould surrounding Camembert cheese was observed with white fungal masses (Figure 2). Fibrin clots and fungal masses were removed, and pathological examination revealed numerous *Aspergillus* hyphae (Figure 3). His symptoms improved after surgery.

**FIGURE 1:**
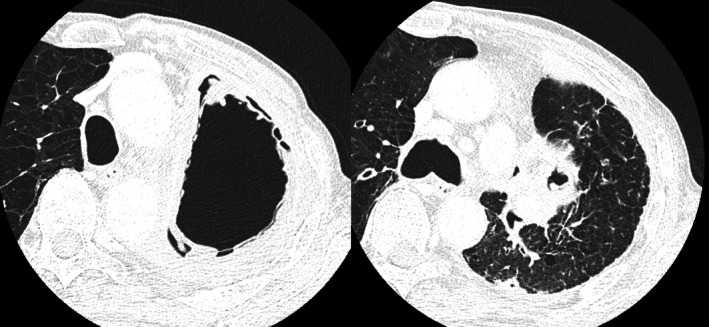
Chest computed tomography demonstrating a postoperative thoracic cavity in the upper left hemithorax with irregular inner wall thickening and communication between the bronchus and the cavity.

**FIGURE 2:**
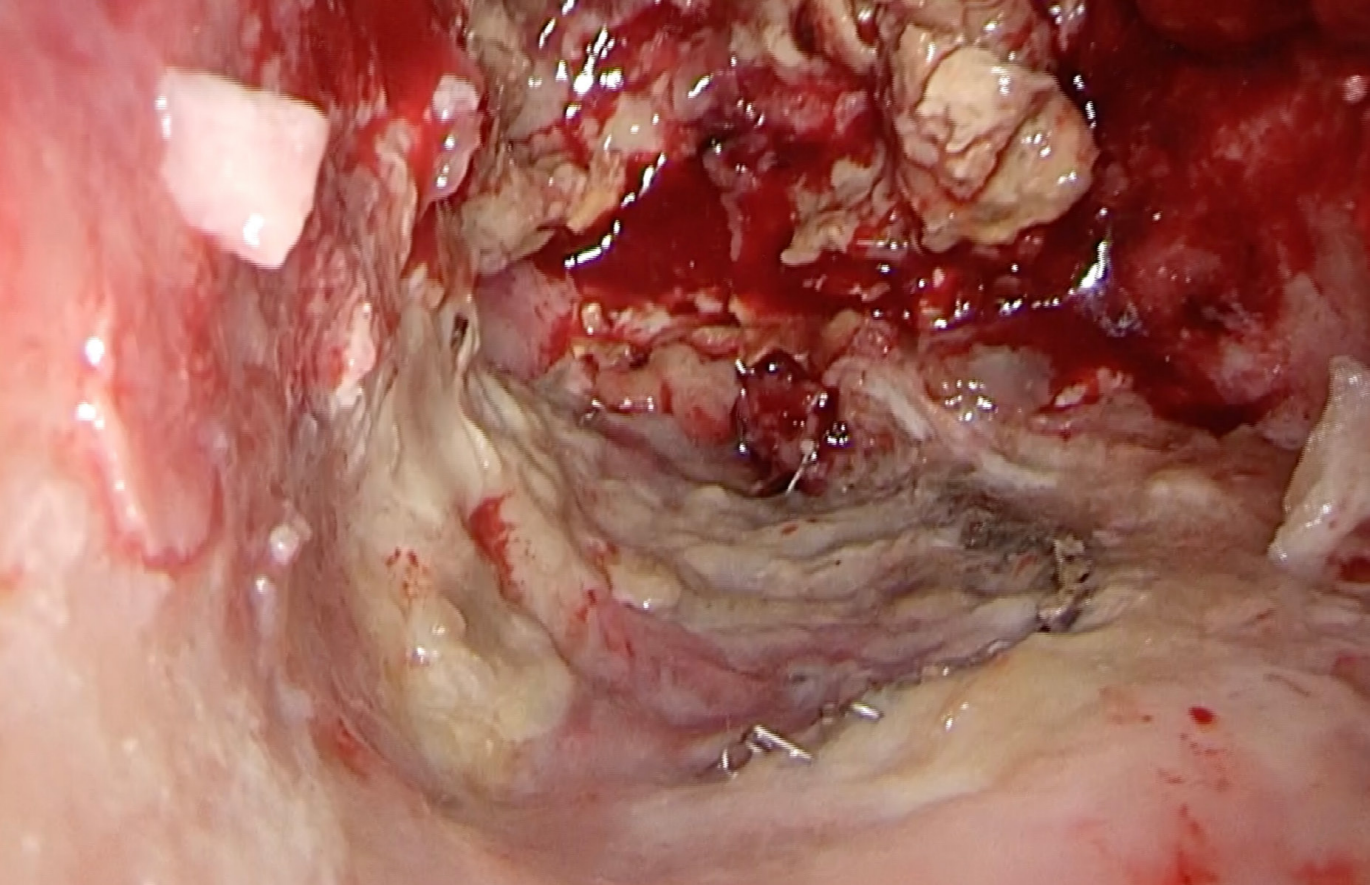
Intraoperative view obtained during fenestration showing purulent pleural debris and white fungal masses within the cavity.

**FIGURE 3:**
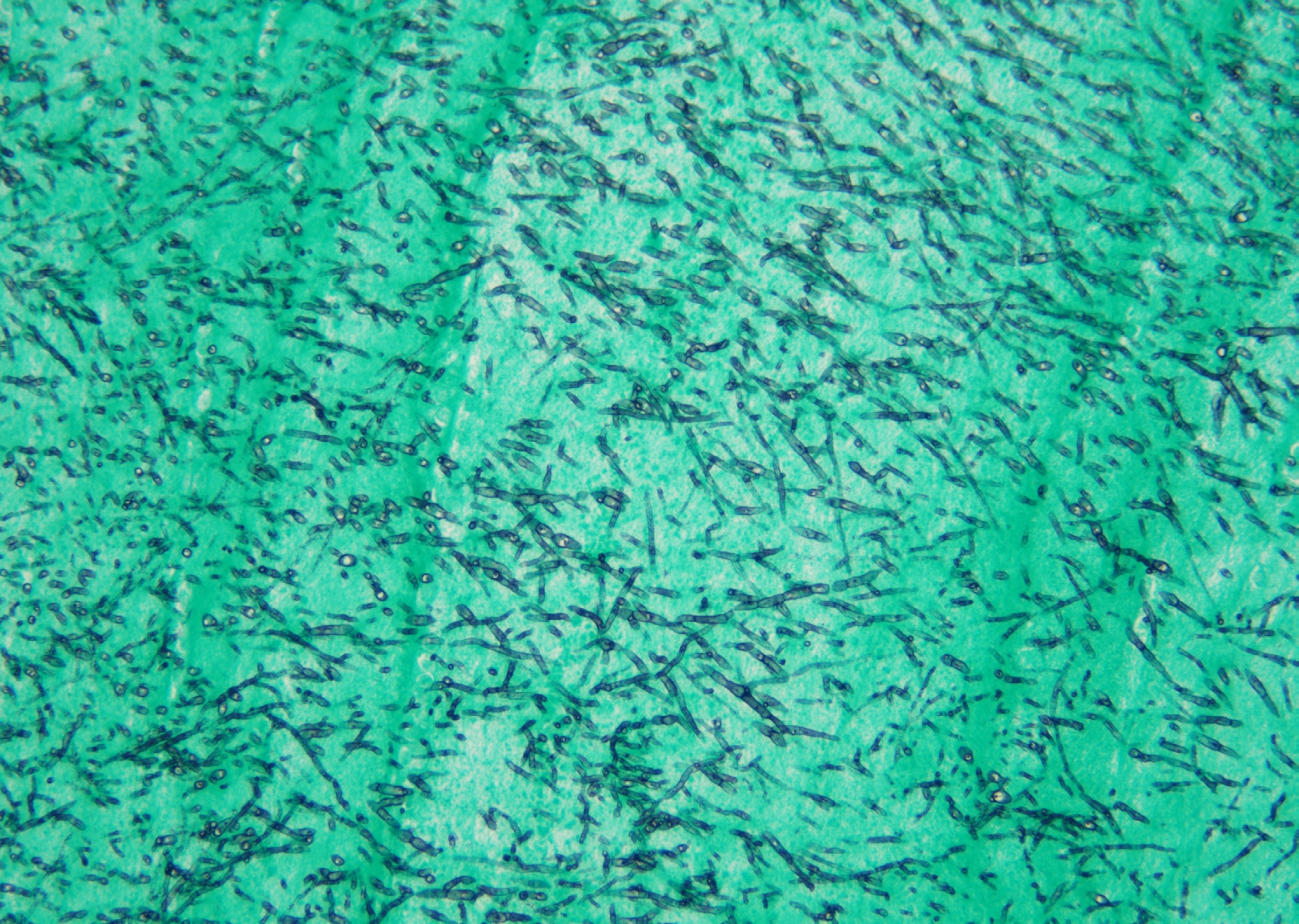
Histopathological examination of the surgical specimen demonstrates numerous *Aspergillus* hyphae with positive Grocott staining.

*Aspergillus* species preferentially colonize pre‐existing structural lung abnormalities [1]. In this case, infection developed not within the lung parenchyma but within a postoperative residual thoracic cavity. The infection formed an inner layer along the cavity surface, and fenestration allowed direct visualization of purulent pleural debris within the cavity. *Aspergillus* pleurisy has been reported in association with bronchopleural fistula, with prior lung resection recognized as a risk factor [2]. Postoperative smoking may have increased susceptibility to infection in this case.

## Author Contributions

Mio Sugino drafted the manuscript. Yusaku Kusaba, Masaaki Nagano and Shinyu Izumi revised the manuscript. All authors approved the final manuscript.

## Consent

The authors declare that written informed consent was obtained for the publication of this manuscript and accompanying images and attest that the form used to obtain consent from the patient complies with the Journal requirements as outlined in the author guidelines.

## Conflicts of Interest

The authors declare no conflicts of interest.

## Data Availability

The data that support the findings of this study are available on request from the corresponding author. The data are not publicly available due to privacy or ethical restrictions.
